# Anterior neck soft tissue measurements on computed tomography to predict difficult laryngoscopy: a retrospective study

**DOI:** 10.1038/s41598-021-88076-z

**Published:** 2021-04-19

**Authors:** Hye Jin Kim, Nar Hyun Min, Jong Seok Lee, Wootaek Lee, Do-Hyeong Kim

**Affiliations:** Department of Anesthesiology and Pain Medicine, Anesthesia and Pain Research Institute, Yonsei University College of Medicine, 50-1 Yonsei-ro, Seodaemun-gu, Seoul, 03722 Republic of Korea

**Keywords:** Medical research, Risk factors

## Abstract

Predicting difficult laryngoscopy is an essential component of the airway management. We aimed to evaluate the use of anterior neck soft tissue measurements on computed tomography for predicting difficult laryngoscopy and to present a clear measurement protocol. In this retrospective study, 281 adult patients whose tracheas were intubated using a direct laryngoscope for thyroidectomy were enrolled. On computed tomography, the distances from the midpoint of the thyrohyoid membrane to the closest concave point of the vallecular (membrane-to-vallecula distance; dMV), and to the most distant point of the epiglottis (membrane-to-epiglottis distance; dME) were measured, respectively. The extended distances straight to the skin anterior from the dMV and dME were called the skin-to-vallecula distance (dSV) and skin-to-epiglottis distance (dSE), respectively. Difficult laryngoscopy was defined by a Cormack-Lehane grade of > 2. Difficult laryngoscopy occurred in 40 (14%) cases. Among four indices, the dMV showed the highest prediction ability for difficult laryngoscopy with an area under the receiver operating characteristic curve of 0.884 (95% confidence interval 0.841–0.919, *P* < 0.001). The optimal dMV cut-off value for predicting difficult laryngoscopy was 2.33 cm (sensitivity 75.0%; specificity 93.8%). The current study provides novel evidence that increased dMV is a potential predictive indicator of difficult laryngoscopy.

## Introduction

Difficult laryngoscopy is still reported in 5–18% of patients despite the incessant development of airway devices, techniques and protocol revisions^1–4^. This can lead to fatal complications, such as death and brain damage, with a reported incidence of 1 per 110,000 cases in the UK, according to the 4th National Audit Project of the Royal College of Anaesthetists' and Difficult Airway Society reports^5^. Therefore, it is crucial to predict difficult laryngoscopy and make thorough preparations to minimise the detrimental outcomes.

Known screening tests for difficult laryngoscopy include neck mobility assessment, upper lip bite test, inter-incisor distance, thyromental distance and modified Mallampati score, amongst others. However, the predictive power, sensitivity and specificity of each screening test is poor, and even if multiple tests are combined, the predictive power is not satisfactory^3,6^. This implies that there is a great need for a new screening test.

Recently, imaging modalities have broadened of the scope of research on predicting difficult laryngoscopy, and one meta-analysis even showed the superior diagnostic value of radiologic measurements compared to previously known clinical screening tests^7^. In a pilot study, sonographic measurement of anterior neck soft tissue (ANS) showed high predictive power but low correlation with the previous screening test, implying that new methods may be able to fill the gaps present in existing prediction models^8^. Moreover, subsequent studies^2,9,10^ reported the moderate-to-high predictive power of sonographic ANS measurements, such as pre-epiglottic space (PES) depth, and distance from skin to the epiglottis or trachea. However, cut-off values and level of measurement (vocal cord, thyrohyoid membrane) were inconsistent and contradictory in these studies. Different protocols of measurement between studies and innate problems of ultrasound-based research, i.e., validity and reliability^11^, could be possible explanations. Ultrasound is excellent in terms of economic efficiency and accessibility, but less reliable in measuring the depth of the epiglottis compared to computed tomography (CT)^12,13^.

Therefore, we selected CT to obtain accurate measurements, and to select distinct anatomical landmarks on the CT images on which, these measurements can be based. In this retrospective study, we aimed to evaluate the power of ANS measurements on CT scans to predict difficult laryngoscopy and to present a clear measurement protocol with designated landmarks. To do this, we chose the depth of PES and the depth of the epiglottis.

## Methods

### Patients

The Institutional Review Board of Gangnam Severance Hospital (IRB no. 3–2020-0129 on May 2020) approved this study and waived the requirement for written informed consent. This retrospective study was conducted in accordance with the Declaration of Helsinki. Data were retrospectively collected from the electronic medical records of patients who had undergone elective thyroidectomy for suspected thyroid malignancy at Gangnam Severance Hospital, Yonsei University Health System, Seoul, Korea from April 2019 to March 2020. We chose this study population because most of them had preoperative neck CT images. We included patients aged ≥ 19 years with American Society of Anaesthesiologists (ASA) physical status of I–III and whose tracheas were intubated using direct laryngoscopy. Patients without available CT images (both axial and sagittal views) in the picture archives and communication system were excluded. In addition, patients whose imaging results did not contain information on CT indicators analysed in this study, were excluded.

### CT image analysis

Neck CT scans were obtained with the patient supine, and the head and neck in a neutral position supported by a head rest cushion (part number: 7445740; Siemens GE Healthcare, Milwaukee, WI, USA). CT measurements were taken from the sagittal sections on a picture archiving and communication system viewer (Centricity; General Electric Medical Systems, Milwaukee, WI, USA).

In the axial view, the midline at the vallecula level was selected as the reference line, and the corresponding sagittal view was observed. The centre of the imaginary line between the lower margin of the hyoid bone where the thyrohyoid membrane starts and the laryngeal prominence of the thyroid cartilage where it ends was assumed to be the midpoint of the thyrohyoid membrane.

**Figure 1:**
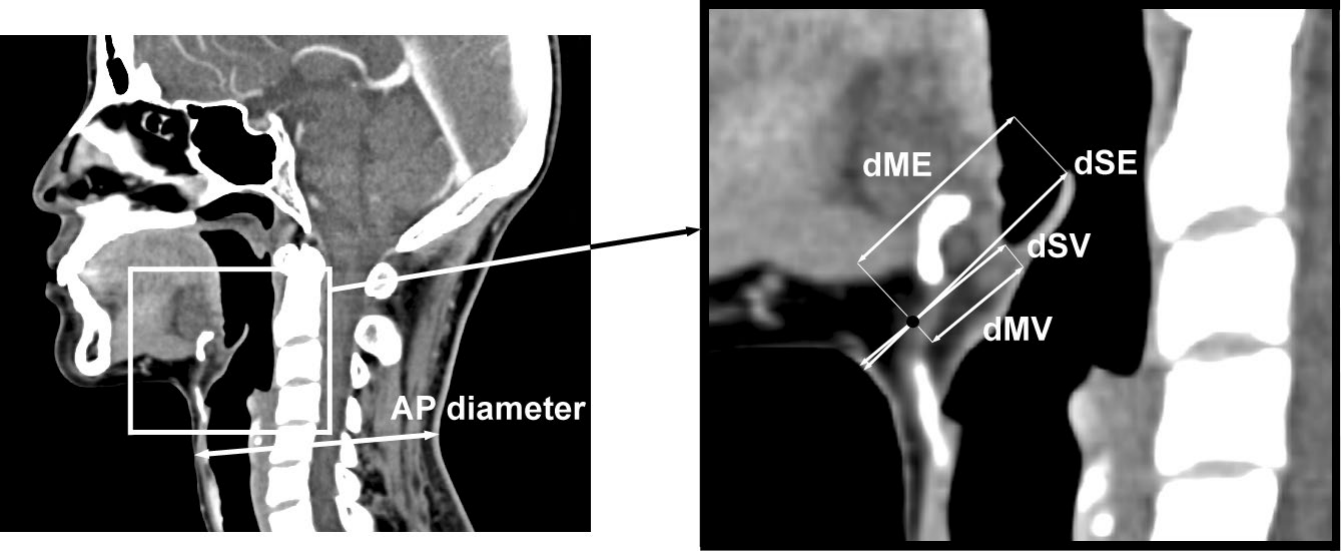
Anterior neck measurements. The picture on the right is an enlarged image of the white box on the picture on the left. The neck anterior–posterior (AP) diameter is the shortest AP distance that passes the centre of the cricoid cartilage on the lowest axial section of the cricoid. The membrane-to-vallecula distance (dMV) is the distance from the midpoint of the thyrohyoid membrane between the hyoid bone and laryngeal prominence to the closest concave point of the vallecula. The membrane-to-epiglottis distance (dME) is the distance from the midpoint of the thyrohyoid membrane between the hyoid bone and laryngeal prominence to the furthermost tip of the epiglottis. The skin-to-vallecula distance (dSV) is the extended distance straight to the skin anterior from the dMV. The skin-to-epiglottis distance (dSE) is the extended distance straight to the skin anterior from the dME.

The distance from the midpoint of the thyrohyoid membrane to the closest concave point of the vallecula, and the distance from the midpoint of the thyrohyoid membrane to the most distant point of the epiglottis were called the membrane-to-vallecula distance (dMV) and the membrane-to-epiglottis distance (dME), respectively (Fig. 1). In addition, the extended distances straight to the skin anterior from the dMV and dME were called the skin-to-vallecula distance (dSV) and the skin-to-epiglottis distance (dSE), respectively (Fig. 1). Both dMV and dSV reflected the thickness of the PES, while dME and dSE reflected the depth of the epiglottis. Two staff anaesthesiologists measured each parameter, and the average value was used for the analysis.

The neck anterior–posterior (AP) diameter (Fig. 1) was measured as a substitute for the neck circumference because the axial section of the CT image taken in the supine position was not suitable for measuring the neck circumference. The positional relationship between the mandible and pharynx in the sagittal plane of the CT image is imprecise, as the AP diameter at the thyroid cartilage level often appears increased due to the overlap of the lower jaw, which can be solved if the patient raises his or her head in the erect position. Therefore, the lowest axial section of the cricoid^14^ was used as the measurement standard, and the shortest line that touched the skin at the front of the neck past the centre of the cricoid cartilage was drawn and connected to the posterior neck skin.

### Anaesthesia and data collection

Baseline patient demographic data were extracted from the medical records including age, sex, height, weight, body mass index (BMI) and ASA class. Resident anaesthesiologists performed preoperative airway evaluation, including evaluation of the modified Mallampati score, inter-incisor distance and thyromental distance. The modified Mallampati score was evaluated while the patient was sitting without phonation. Inter-incisor distance was measured when the mouth was maximally open, and the thyromental distance was measured with the patient’s head fully extended and mouth closed. All measurements were performed using a ruler.

Upon arriving at the operating room, the patient was placed under standard monitors, which evaluated pulse oximetry, 3-lead electrocardiography and non-invasive blood pressure monitoring. Anaesthesia was induced using 1–2 mg/kg of propofol and 0.5–1.0 µg/kg of remifentanil. After confirming the loss of consciousness, positive pressure ventilation was provided via mask bagging. Rocuronium bromide 0.6–1.2 mg/kg was administered to induce muscle relaxation and a peripheral nerve stimulator (Innervator 252; Fisher & Paykel Healthcare, Auckland, New Zealand) was used to check for adequate relaxation. The absence of response to 50 Hz train-of-four stimulation on the ulnar nerve at the adductor pollicis muscle, using this tool, was considered adequate for intubation. With the patient in a supine position with a pillow under the head, tracheal intubation was performed by senior anaesthesia residents or fellows with at least 3 years of experience using a direct laryngoscope. For unanticipated difficult airways, our institution followed the 2015 Difficult Airway Society guidelines^15^.

Anaesthesia records were obtained, which included intubating provider; modified Cormack-Lehane grade; application of the backward, upward and rightward pressure manoeuvre; intubation failure; and occurrence of complications (teeth damage, bleeding from oropharynx). The Cormack-Lehane grade was recorded before applying the backward, upward, rightward pressure manoeuvre as per our institutional practice.

The definition of difficult laryngoscopy was a Cormack-Lehane grade of more than two.

### Study endpoint

The primary endpoint was the predictive value of each ANS measurement, i.e., dMV, dME, dSV and dSE acquired by CT scan, in predicting difficult laryngoscopy using the direct laryngoscope.

### Statistical analysis

Data analysis was performed in patients with complete ANS measurements and available Cormack-Lehane grades. Patients were divided into two groups based on whether they had a difficult laryngoscopy. After conducting the Shapiro–Wilk test for normality, continuous variables were analysed using the independent t-test or Mann–Whitney U test. Categorical variables were analysed by Chi-square test or Fisher’s exact test. Binary data were presented as numbers (%), whereas continuous data were presented as mean (standard deviation) if normally distributed or as median [interquartile range] if otherwise. The area under the receiver operating characteristic (AUROC) curve was calculated to measure the capability of the ANS indices (dMV, dME, dSV and dSE) to predict difficult laryngoscopy. The optimal cut-off value was determined by maximising the Youden index. Using bootstrap methodology with 1000 multiple samples, 95% confidence intervals (CI) of the best threshold were determined as the grey zone. Among ANS indices, the most significant indicator was analysed separately for men and women. After checking for multicollinearity, multivariable logistic regression analysis was performed through backward stepwise selection to assess the impact of age, modified Mallampati score, inter-incisor distance, neck AP diameter and one of the four ANS indices with the highest AUROC on the occurrence of difficult laryngoscopy, adjusted for sex. Models were compared based on their Akaike’s information criterion. Statistical significance was set at *P* < 0.05. Inter-observer reproducibility was assessed in all datasets for ANS indices. This was done by calculating an intraclass correlation coefficient and a coefficient of variation. Inter-observer agreement for the estimation of the ANS index was tested using a Bland–Altman plot.

All analyses were performed using the Statistical Package for the Social Sciences Statistics for Windows, version 25 software (IBM Corp., Armonk, NY, USA), R version 3.5.3 (The R Foundation for Statistical Computing, Vienna, Austria) and MedCalc version 19.5.1 (MedCalc, Ostend, Belgium). Figures 3 and 4 were created using MedCalc version 19.5.1 (MedCalc, Ostend, Belgium; https://www.medcalc.org).

### Sample number calculation

Assuming that the number of predictors were three or four based on the logistic regression analysis, the number of difficult laryngoscopies required should at least be between 30 and 40 according to the event-per-variable rule. We assumed that the incidence of difficult laryngoscopy is about 10–15%^1,3^; therefore, to obtain 30–40 cases of difficult laryngoscopy, 200–400 intubation cases were needed.

Of the patients who underwent elective thyroidectomy performed in April 2019 at our institution, there were 25 cases of endotracheal intubation using direct laryngoscope. Assuming approximately 25 cases per month, the expected number of cases per year was approximately 300.

## Results

**Figure 2:**
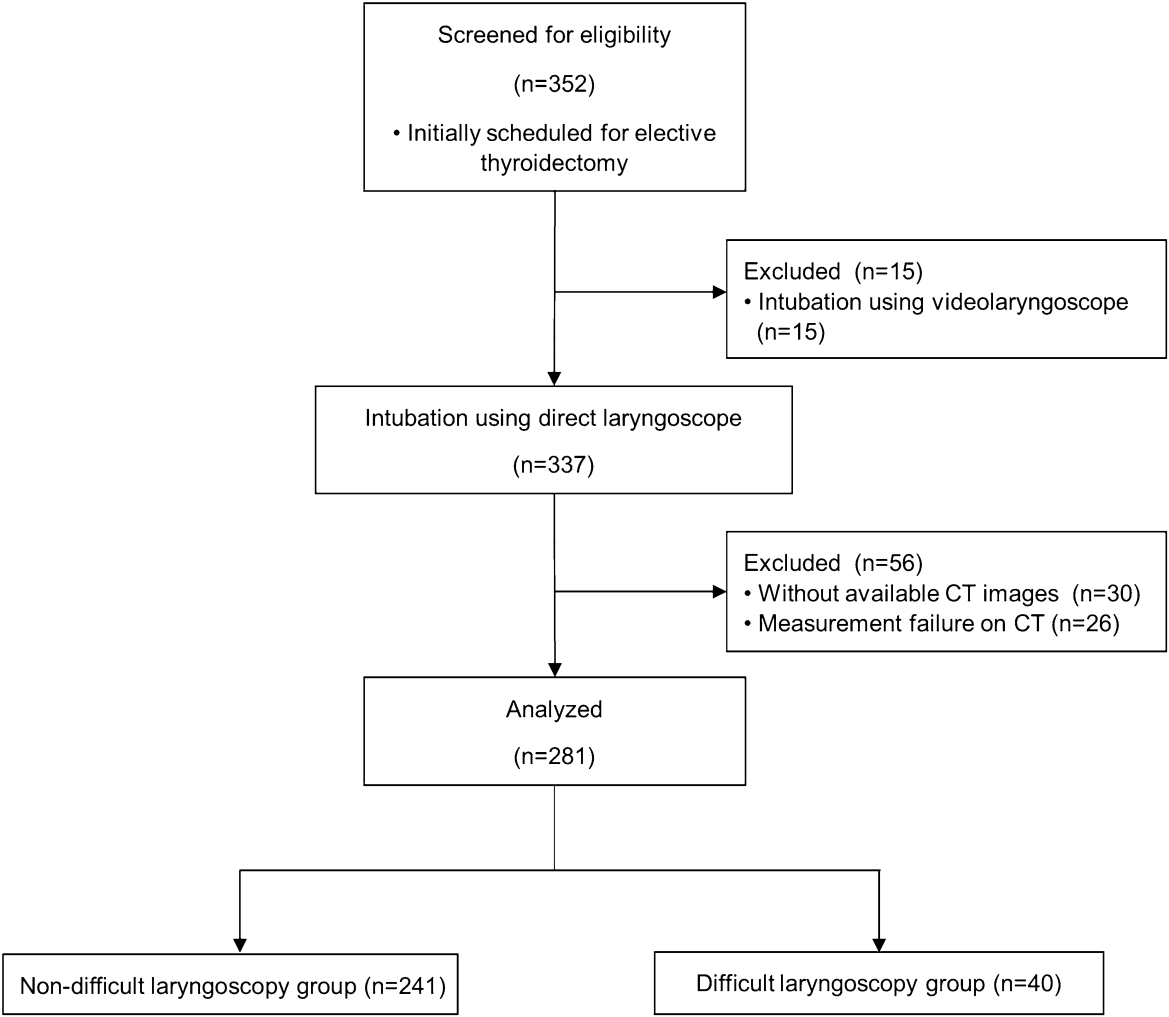
Flow chart of the selection of the study population. Among the 352 patients who were scheduled for elective thyroidectomy from April 2019 to March 2020 at our institution, 15 patients whose tracheas were intubated using video-laryngoscope are excluded. Among the remaining 337 patients, 30 patients without available computed tomography scan images (both axial and sagittal views) and 26 patients in which measurement of the vallecula was not possible are excluded. Finally, 281 patients with complete anterior neck soft tissue measurements and available records of Cormack-Lehane grade are included. One patient from the non-difficult laryngoscopy group is excluded from the area under the receiver operating characteristic curve calculation for neck AP diameter and the logistic regression analysis because of missing neck AP diameter data due to the impossibility of measurements owing to a tracheal deviation by mass.

We initially screened 352 patients who were scheduled for thyroidectomy. Among them, 71 patients were excluded for the reasons mentioned in the Methods section, and finally, 281 patients were included (Fig. 2). One patient had missing neck AP diameter data, which was impossible to measure because of tracheal deviation by the mass, and this patient was excluded from the AUROC curve calculation for the neck AP diameter and the logistic regression analysis. Difficult laryngoscopy occurred in 40 patients (14%).

Baseline patient characteristics, including sex, height, weight, BMI, ASA class and thyromental distance were not significantly different between the non-difficult laryngoscopy and difficult laryngoscopy groups (Table 1). Patients in the difficult laryngoscopy group were older, with higher modified Mallampati score (3 [3–3] vs. 2 [1–2], *P* < 0.001) and shorter inter-incisor distance. The means of the dMV, dME, dSV and dSE were higher in patients in the difficult laryngoscopy group (*P* < 0.001 for up to the third variable, *P* = 0.003 for dSE). Moreover, neck AP diameter was greater in patients in the difficult laryngoscopy group (*P* = 0.001). The backward, upward, rightward pressure manoeuvre was performed more frequently in the difficult laryngoscopy group. Dental damage and bleeding from the oropharynx occurred simultaneously in only one person in the non-difficult laryngoscopy group, and there was no significant difference in occurrence of complications between the two groups (*P* > 0.999).

**Table 1:** Demographic characteristics, airway parameters and intubation data in the non-difficult laryngoscopy and difficult laryngoscopy groups.

	Non-difficult laryngoscopy (n = 241)	Difficult laryngoscopy (n = 40)	*P* value
**Demographic characteristics**			
Age (years)	43.0 ± 12.3	47.2 ± 12.8	0.049
Sex, male	63 (26.1)	14 (35.0)	0.331
Height (cm)	163.7 ± 8.1	164.8 ± 7.9	0.407
Weight (kg)	63.8 ± 12.5	66.9 ± 10.9	0.137
BMI (kg m^−2^)	23.7 ± 3.6	24.6 ± 3.3	0.141
ASA physical status 1/2/3	128/98/15	18/17/5	0.307
**Airway parameter**			
Modified Mallampati classification			< 0.001
1	77 (32.0)	1 (2.5)	
2	108 (44.8)	7 (17.5)	
3	52 (21.6)	28 (70.0)	
4	4 (1.7)	4 (10.0)	
Inter-incisor distance (cm)	4.9 ± 1.0	4.4 ± 0.8	0.001
Thyromental distance (cm)	8.3 ± 1.5	8.1 ± 1.4	0.455
dMV (cm)	1.9 ± 0.3	2.5 ± 0.5	< 0.001
dME (cm)	3.4 ± 0.5	3.7 ± 0.5	< 0.001
dSV (cm)	3.2 ± 0.7	3.9 ± 0.8	< 0.001
dSE (cm)	4.7 ± 0.7	5.0 ± 0.7	0.003
Neck AP diameter (cm)*	11.0 ± 1.2	11.7 ± 1.3	0.001
**Intubation data**			
Intubating provider			0.203
Senior residents	233 (92.5)	34 (85.0)	
Fellows	18 (7.5)	6 (15.0)	
Cormack-Lehane grade	101/140/0/0	0/0/38/2	< 0.001
BURP manoeuvre	53 (22.0)	37 (92.5)	< 0.001
Intubation failure	0 (0.0)	0 (0.0)	> 0.999
Complications	1 (0.4)	0 (0.0)	> 0.999

Data are presented as mean (standard deviation), number, or number (proportion).

AP, anterior–posterior; ASA, American Society of Anaesthesiologists; BMI, body mass index; BURP, backward, upward, rightward pressure; dME (membrane-to-epiglottis distance), distance from the midpoint of thyrohyoid membrane between the hyoid bone and laryngeal prominence to the furthermost tip of the epiglottis; dMV (membrane-to-vallecula distance), distance from the midpoint of the thyrohyoid membrane between the hyoid bone and laryngeal prominence to the closest concave point of the vallecula; dSE (skin-to-epiglottis distance), extended distance straight to the skin anterior from the dME; dSV (skin-to-vallecula distance), extended distances straight to the skin anterior from the dMV.

*One patient from the non-difficult laryngoscopy group is excluded because of missing neck AP diameter data due to the impossibility of measurements owing to a tracheal deviation by mass.

**Figure 3:**
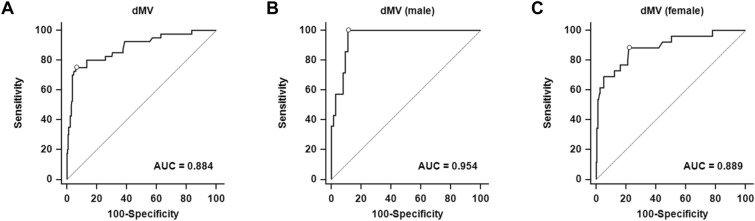
Receiver operating characteristic curves showing the ability of the membrane-to-vallecula distance (dMV) to predict difficult laryngoscopy**.** From left to right, the area under the curve for the dMV is 0.884 (95% CI 0.841–0.919) for both sexes (**A**), 0.954 (95% CI 0.880–0.988) in men (**B**) and 0.889 (95% CI 0.837–0.928) in women (**C**). The diagonal line indicates 50% discrimination and is provided to show how much each model improves on purely random assignment of the risk. CI indicates confidence interval.

The receiver operating characteristic curve analysis (Fig. 3 and Table 2) showed that the AUROC curve for the dMV was 0.884 (95% CI 0.841–0.919, *P* < 0.001), which represented the highest prediction ability among ANS indices. The optimal dMV cut-off value for predicting difficult laryngoscopy was 2.33 cm (sensitivity 75.0%; specificity 93.8%).

**Table 2:** Prediction of difficult laryngoscopy by the receiver operating characteristic curves of the airway parameters.

	AUROC curve (95% CI)	*P* value	Optimal cut-off value*	Grey zone†	Patients in grey zone (%)	Sensitivity (%) (95% CI)	Specificity (%) (95% CI)	Youden Index (J)
Modified Mallampati classification	0.811 (0.760–0.855)	< 0.001	> 2	–	–	80.0 (64.4–90.9)	76.8 (70.9–81.9)	0.568
Inter-incisor distance (cm)	0.658 (0.600–0.714)	< 0.001	≤ 4.5	4.4–5.5	135 (48.0)	70.0 (53.5–83.4)	60.2 (53.7–66.4)	0.302
Thyromental distance (cm)	0.553 (0.493–0.612)	0.263	≤ 8.5	6.5–10.2	234 (83.3)	70.0 (53.5–83.4)	43.6 (37.2–50.1)	0.136
dMV (cm)	0.884 (0.841–0.919)	< 0.001	> 2.33	1.94–2.37	91 (32.4)	75.0 (58.8–87.3)	93.8 (89.9–96.5)	0.688
dMV (cm) (men)	0.954 (0.880–0.988)	< 0.001	> 2.37	2.36–2.73	13 (16.9)	100 (76.8–100.0)	88.9 (78.4–95.4)	0.889
dMV (cm) (women)	0.889 (0.837–0.928)	< 0.001	> 1.94	1.75–2.22	86 (42.2)	88.5 (69.8–97.6)	78.1 (71.3–83.9)	0.666
dME (cm)	0.691 (0.634–0.745)	< 0.001	> 3.27	3.22–4.09	143 (50.9)	87.5 (73.2–95.8)	48.6 (42.1–55.0)	0.361
dSV (cm)	0.742 (0.687–0.792)	< 0.001	> 3.37	3.24–3.55	60 (21.4)	77.5 (61.5–89.2)	65.6 (59.2–71.5)	0.431
dSE (cm)	0.635 (0.576–0.692)	0.003	> 4.54	3.87–5.01	153 (54.4)	80.0 (64.4–90.9)	44.4 (38.0–50.9)	0.244
Neck AP diameter (cm)‡	0.659 (0.600–0.714)	< 0.001	> 11.00	10.10–12.81	185 (65.8)	62.5 (45.8–77.3)	63.3 (56.9–69.4)	0.258

AP, anterior–posterior; AUROC, area under the receiver operating characteristic; CI, confidence interval; dME (membrane-to-epiglottis distance), distance from the midpoint of the thyrohyoid membrane between the hyoid bone and laryngeal prominence to the furthermost tip of the epiglottis; dMV (membrane-to-vallecula distance), distance from the midpoint of the thyrohyoid membrane between the hyoid bone and laryngeal prominence to the closest concave point of the vallecula; dSE (skin-to-epiglottis distance), extended distances straight to the skin anterior from the dME; dSV (skin-to-vallecula distance), extended distances straight to the skin anterior from the dMV.

*Optimal cut-off values are determined by maximising the Youden index.

^†^Grey zone: based on 1000 bootstrap samples, 95% CIs of the best threshold are determined.

^‡^One patient from the non-difficult laryngoscopy group is excluded because of missing neck AP diameter data due to the impossibility of measurements owing to a tracheal deviation by mass.

In terms of sex, the AUROC curve for the dMV was 0.954 (95% CI 0.880–0.988, *P* < 0.001) with 100.0% sensitivity and 88.9% specificity at a cut-off value of 2.37 cm in men, and 0.889 (95% CI 0.837–0.928, *P* < 0.001) with 88.5% sensitivity and 78.1% specificity at a cut-off value of 1.94 cm in women (Fig. 3 and Table 2). In both sexes, predictability and sensitivity increased with the use of the dMV according to sex, but in men, the extent of increase was greater, and the number of patients in the grey zone (16.9%) was almost half that of the average of the total study population (both sexes).

In the multivariable logistic regression analysis adjusted for sex, modified Mallampati score (odds ratio [OR] 3.88, 95% CI 1.80–8.38) and dMV (OR 143.78, 95% CI 27.75–744.89) were independently associated with difficult laryngoscopy, while age, inter-incisor distance and neck AP diameter were not (Table 3).

**Table 3:** Results of logistic regression analysis to predict difficult laryngoscopy, adjusted for sex.

	Odds ratio (95% confidence interval)	*P* value
Modified Mallampati score	3.88 (1.80–8.38)	< 0.001
Inter-incisor distance (cm)	0.58 (0.31–1.08)	0.085
dMV (cm)	143.78 (27.75–744.89)	< 0.001

dMV (membrane-to-vallecula distance), distance from the midpoint of the thyrohyoid membrane between the hyoid bone and laryngeal prominence to the closest concave point of the vallecula.

**Figure 4:**
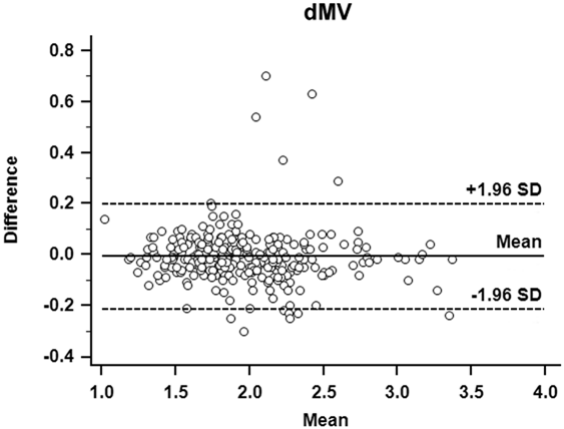
Bland–Altman analysis of the agreement between the investigators for membrane-to-vallecula distance (dMV) measurements. The differences between the investigators are plotted against the averages of the two investigators.

Inter-observer variability for measuring dMV was excellent, with an intraclass correlation coefficient of 0.97 (95% CI 0.96–0.97) and a coefficient of variation of 3.8%. Each inter-observer variability for dME, dSV and dSE were also excellent. Using a Bland–Altman analysis for evaluating inter-observer agreement in estimating the dMV, the mean of the biases was − 0.01 cm (with 95% limits of agreement between − 0.21 and 0.20 cm) (Fig. 4). The remaining indices displayed similar results.

## Discussion

This study showed that increased ANS thickness at the level of the thyrohyoid membrane measured on CT is predictive of difficult laryngoscopy, with dMV showing excellent predictive ability. In addition, dMV and modified Mallampati score were independently associated with difficult laryngoscopy, while dMV showed a slightly higher AUROC curve value than that of the modified Mallampati score although there was no statistically significant difference between them.

The line of the dMV crosses the PES, a fat-containing structure that is bordered superiorly by the hypoepiglottic ligament anterior to the thyrohyoid ligament, and posterior-inferiorly by the epiglottis and thyroepiglottic ligament. These boundaries may limit the mobility and plasticity of the PES that occurs during the neck extension or upward movement of the laryngoscope blade^2^. In other words, as the dMV increases, the room for lifting the vallecula is restricted, and upward movement of the blade is limited. This was a novel finding in this study, which could provide guidance for future research.

On the other hand, the skin in front of the thyrohyoid membrane has excellent mobility and elasticity^16^. Therefore, the pressure of the blade easily causes anterior movement, so skin thickness seems to have a relatively minor effect on difficult laryngoscopy. This might explain the lower predictability of dSV. Furthermore, as mentioned in the Methods section, the overlap of the lower jaw and thyroid cartilage on CT images makes it difficult to measure dSV and dSE, indicating that the skin is an ambiguous landmark. It appears that dMV has higher predictive power than dME given that the epiglottis is indirectly elevated by vallecular lifting. Moreover, neck AP diameter was not an independent predictor of difficult laryngoscopy in our study. Individual variances in fat disposition around the AP neck may contribute to this result. Inconsistent reports on neck circumference as an independent predictor of difficult airway^14,17^ can be considered in the same context. It is interesting that inter-incisor distance was found to not be independently associated with difficult laryngoscopy in this study. This may be explained because our study included only three patients whose inter-incisor distance was less than 3 cm^6^.

It is noteworthy that when dMV cut-off values were applied differently according to sex, predictive power and sensitivity increased in both cases, especially in men. Different anatomic features between the sexes, such as laryngeal prominence, might explain this result and justify the analysis of dMV by sex.

The measurements of ANS thickness was based on the hypothesis that its increase would impede the anterior mobility of the pharyngeal structures^2^. Most previous studies concerning ANS measurement using ultrasound suggest that ANS thickness will affect difficult laryngoscopy; however, these studies showed inconsistent results, most likely due to the differences in the enrolled patient groups, measurement levels and protocols. An initial trial including 50 obese patients (BMI > 35) reported that an abundance of pretracheal soft tissue at the level of the vocal cords was a good predictor of difficult laryngoscopy^1^. Another trial by Reddy et al.^2^ conducted on 100 patients (BMI range 14.2–39.0) supported this result, but showed a cut-off value of 0.23 cm, which was different from the value reported in the initial trial. This might have originated from the difference of the study populations in the two trials, in particular, BMI or patient position during measurement (not disclosed in the initial trial; sniffing position in the latter trial). In contrast to these findings, one pilot study^8^ (n = 51, no disclosure of BMI; neutral position during measurement) failed to prove the predictability of ANS thickness at the level of the vocal cords. Instead, the authors reported ANS width at the level of thyrohyoid membrane as a predictor of difficult laryngoscopy with a cut-off value of 2.8 cm. Moreover, two recent trials^9,10^ (n = 74 and n = 301, respectively, both using a neutral position during measurement) showed consistent findings on the predictability of ANS thickness (skin to epiglottis distance) at the level of the thyrohyoid membrane on difficult laryngoscopy with cut-off values of 2.75 cm and 2.54 cm, respectively. Different exclusion criteria (the former excluded morbidly obese patients; the latter excluded patients in whom difficult airway was anticipated), different definitions of difficult laryngoscopy in the latter study (Cormack-Lehane grade of 2b or more) and inter-variability issues of ultrasound measurements might have contributed to these different cut-off values despite the same trend. Falcetta^10^ speculated that increased ANS thickness at the thyrohyoid membrane level would resist flattening of the primary or oropharyngeal curve by laryngoscopic manipulation, thus disturbing the line of sight with reference to Greenland's two-curve theory. Considering the surface that is subjected to the direct upward movement of the blade of the laryngoscope, measurement of thickness at the thyrohyoid membrane level seems more plausible than at the lower part, i.e., level of the vocal cord.

A strength of this study was that we tried to verify our hypothesis with a clear protocol showing excellent inter-observer variability. Moreover, we chose CT to overcome the shortcomings of ultrasound research. Ultrasound measurement is greatly influenced by the technique and skill level of the operator, and the pressure of the probe^13,18,19^.

Our study had several limitations. First, we did not include the upper lip bite test, neck mobility and neck circumference in the analyses for predicting difficult laryngoscopy as the corresponding records were unavailable, which is a limitation of retrospective studies. To fill these gaps, the neck AP diameter was used as an indirect indicator of the neck circumference. Second, because many patients do not have CT results or their vallecula cannot be seen on the CT image in real practice, the results of this study may be difficult to apply in routine clinical practice. Third, different cut-off values may be drawn for different races due to anatomical differences. However, our introduction of dMV as a reference to predict difficult laryngoscopy may guide future research in various ethnic populations.

In conclusion, the current study provides the novel finding that increased dMV is a potential predictive indicator of difficult laryngoscopy, and provides superior predictive power compared to previous predictive models. Future trials with a prospective design are required to prove the predictive ability of dMV.

## Data Availability

The datasets generated during and/or analysed during the current study are available from the corresponding author on reasonable request.
